# Thermal and Mechanical Performance of Maleic Anhidride/Benzoyl Peroxide-Modified PLA/PCL Biocomposites

**DOI:** 10.3390/polym17182540

**Published:** 2025-09-19

**Authors:** Aritz Unamuno Garay, Alexandra Llidó Barragán, Santiago Ferrandiz-Bou, Maria Dolores Samper

**Affiliations:** Department of Mechanical and Materials Engineering, Universitat Politècnica de València (UPV), Plz. Ferrándiz y Carbonell, s/n, 03801 Alcoy, Spain; alllibar@epsa.upv.es (A.L.B.); sferrand@mcm.upv.es (S.F.-B.)

**Keywords:** PLA/PCL blend, maleic anhydride, benzoyl peroxide, compatibilizers

## Abstract

This study investigated PLA/PCL blends modified with maleic anhydride (MA) via radical grafting using benzoyl peroxide (BPO) as an initiator. Different formulations with 5 and 10 wt.% of PLA-g-MA (containing 1, 3, and 5 wt.% MA) were prepared to evaluate their compatibilizing effect. Samples were characterized thermally, mechanically, and morphologically using DSC, TGA, FTIR, goniometry, SEM, and tensile, impact, and hardness tests. The results show that adding PCL significantly improves the ductility of PLA, though it reduces tensile strength and hardness. Grafting with MA partially improves phase compatibility, as seen by increased elongation at break and impact resistance, especially at intermediate MA concentrations (1–3%). However, higher MA contents lead to greater variability in thermal and mechanical results, likely due to heterogeneous phase dispersion. FTIR analysis detected residual BPO in some formulations, though below 0.1 phr. TGA indicated a slight improvement in thermal stability at 5 wt.% MA. Overall, the findings suggest that controlled use of MA as a compatibilizer enhances the balance of mechanical and thermal properties in PLA/PCL systems.

## 1. Introduction

Polymeric materials are widely utilized in modern society due to their excellent mechanical properties, chemical stability, and cost-effectiveness. However, the extensive use of petroleum-based, non-biodegradable polymers has raised serious environmental concerns due to their persistence and accumulation in ecosystems. Consequently, there has been increasing interest in the development of biodegradable and renewable alternatives in both research and industry. Among these, polylactic acid (PLA) has emerged as a promising thermoplastic, valued for its biodegradability, high tensile strength, biocompatibility, and ease of processing [1]. Derived from renewable resources such as corn, wheat, and potatoes, PLA offers a viable replacement for conventional plastics across diverse applications.

Despite its advantages, PLA suffers from inherent drawbacks including brittleness, low impact resistance, and limited flexibility—mainly due to its low crystallinity and rigid molecular structure. To overcome these limitations, several modification strategies have been explored, including physical blending [2], chemical copolymerization [3], and polymer grafting. In particular, blending PLA with more ductile polymers such as poly(ε-caprolactone) (PCL) [4], thermoplastic polyurethane (TPU) [5], polybutylene succinate (PBS) [6], and polybutylene adipate terephthalate (PBAT) [7] has proven effective in enhancing toughness.

Among compatibilization strategies, the chemical grafting of maleic anhydride (MA) onto PLA to form PLA-g-MA has shown high potential to improve interfacial adhesion and dispersion in immiscible blends. This functionalization typically involves the melt blending of PLA with MA and a free radical initiator such as benzoyl peroxide (BPO) or dicumyl peroxide (DCP) at temperatures between 120 and 200 °C [8]. The presence of MA facilitates the formation of reactive anhydride groups along the PLA chain, which significantly enhances the interaction with PCL [9]. However, the grafting process also tends to reduce PLA’s molecular weight due to chain scission, which can influence both mechanical and thermal performance [10,11].

In PLA/PCL systems, immiscibility remains a challenge. Grafting agents such as PLA-g-MA and PLA-g-GMA have demonstrated improved elongation at break and impact strength, with optimized results often found at around 10 wt.% loading [12,13,14]. Gardella et al. reported the best improvement in elongation at break when only 10% of PLA was substituted with PLA-g-MA, while higher contents led to diminishing returns [13]. Similarly, reactive compatibilization using GMA enhanced elongation and impact toughness up to an optimal concentration (3 wt.%), after which performance declined [15]. Morphological observations using SEM often reveal a reduction in PCL particle size and better dispersion in the PLA matrix when compatibilizers are introduced [16].

Commercial compatibilizers such as Elvaloy PTW, containing epoxy and acrylate functionalities, have also proven effective in enhancing impact resistance and ductility in PLA/PCL blends [13]. Studies have shown that PLA/PCL blends compatibilized with Elvaloy PTW at 6 wt.% reached an optimal balance between toughness and stiffness [13]. Additionally, block copolymers like PLLA-b-PCL and PEG-PPG have been shown to reduce PCL particle size and enhance flexibility and elongation at break by up to 25-fold over neat blends [17,18].

Notably, PLA/PCL blends have attracted interest in biomedical and packaging applications due to their tunable degradation rate, shape memory effect, and improved biocompatibility when compatibilized [19]. Although pure MA is not biocompatible, its grafted form (e.g., PLA-g-MA) demonstrates significantly reduced cytotoxicity and becomes suitable for biomedical use [20].

The innovation of this work lies in the systematic evaluation of different grafting levels (1, 3, and 5 wt.%) combined with two compatibilizer contents (5 and 10 wt.%), using benzoyl peroxide (BPO) as an initiator, and assessing their effects not only on thermal and mechanical behavior but also on morphology and surface properties. This integrated and comparative approach allowed us to identify both the beneficial effects and the limitations of over-compatibilization, providing new insights and practical guidelines for optimizing PLA/PCL systems. In this context, the present study aims to investigate the thermal and mechanical performance of PLA/PCL blends compatibilized via maleic anhydride grafting, where different PLA-g-MA loadings (5 and 10 wt.%) with MA concentrations ranging from 1 to 5 wt.% are synthesized and evaluated through FTIR, DSC, TGA, SEM, goniometry, and mechanical testing. The results are expected to deepen the understanding of compatibilization mechanisms and contribute to the optimization of biodegradable high-performance PLA/PCL-based materials.

## 2. Materials and Methods

### 2.1. Materials

In this study, Poly (lactic acid), PLA commercial grade Biopolymer 2003D, was supplied by Nature Works LLC (Minnetonka, MN, USA) in pellet form with a density of 1.24 g cm^−3^. In this study, poly(ε-caprolactone) Capa™ 6800 was used. It was supplied by Perstorp UK Ltd. (Warrington, UK) and had a density of 1.15 g/cm^3^ and a melt flow index (MFI) of 2–4 g/10 min (at 160 °C, under a 2.16 kg load). In addition, maleic anhydride (MA) (99% for synthesis), and Luperox^®^A75 Benzoyl peroxide (BPO) (with 25% H_2_O for synthesis) were supplied by Sigma Aldrich (Madrid, Spain).

### 2.2. Sample Preparation

Prior to sample preparation for processing, PLA and PCL were dried at 50 °C for 24 h in a CARBOLITE oven to eliminate residual moisture.

**Figure 1:**
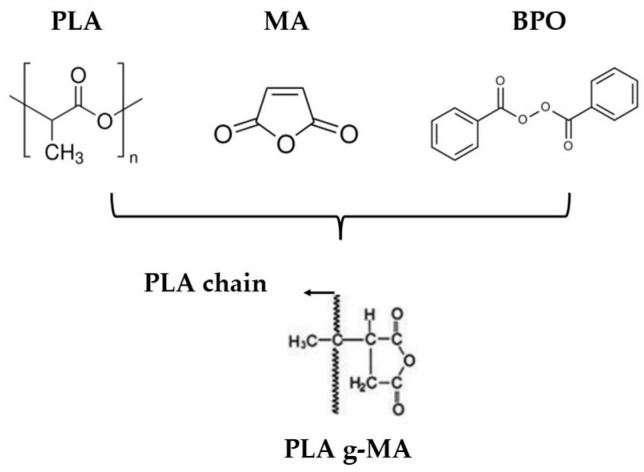
Reaction scheme of polylactic acid, maleic anhydride, and benzoyl peroxide to form grafted maleic anhydride polylactic acid.

After drying, first the PLA grafted with maleic anhydride (MA) was prepared following the compositions outlined in Table 1. Each formulation was subsequently extruded using a twin-screw extruder, model “MC 15 HT,” manufactured by “Xplore” (Sittard, The Netherlands). The extrusion temperature was set at 185 °C, and the screw rotation speed was 100 rpm. The temperature profile was configured as follows: 180 °C (hopper), 185 °C, and 190 °C (die). Before extrusion, a 2 min waiting period was applied to ensure optimal homogeneity of the mixture and to ensure the reaction. Afterwards, these PLA-grafted samples were mechanically grinded. The reaction scheme can be seen in Figure 1.

Secondly, the PLA/PCL blends were fabricated following the compositions outlined in Table 2. Each formulation was subsequently extruded using a twin-screw extruder, model “MC 15 HT,” manufactured by “Xplore” (Sittard, The Netherlands). The extrusion temperature was set at 180 °C, and the screw rotation speed was 100 rpm. The temperature profile was configured as follows: 170 °C (hopper), 175 °C, 180 °C, and 185 °C (die). Before extrusion, a 1 min waiting period was applied to ensure optimal homogeneity of the mixture.

### 2.3. Sample Characterization

#### 2.3.1. Thermal Characterization

Thermal analysis techniques are widely used in the characterization of materials. Differential scanning calorimetry (DSC) was performed in a DSC25 Discovery Series differential scanning calorimeter from TA Instruments (New Castle, DE, USA). Prior to the test, the samples were dried, and the test was conducted in hermetic capsules using a nitrogen environment with a 50 mL/min flow rate to avoid moisture interference. Each sample consisted of between 5 and 6.5 mg and was first heated from 30 to 200 °C at 10 °C·min^−1^, cooled to 30 °C at 10 °C·min^−1^, and again heated from 30 to 200 °C at 10 °C·min^−1^ under inert gas (N_2_). For data analysis, the values obtained from the second heating run were considered, as this eliminates the influence of processing and thermal history, ensuring more reliable and comparable results. Still, the percentage of crystallinity (Xc) is not obtained directly from the DSC curve, but must be calculated using the following equation [21]:

$$Xc=\;\frac{{∆H}_{m}\;\times \;{∆H}_{cc}}{fx\;\;{∆H}_{m}^{0}}\;\times \;100$$

To obtain the mass percentage during the material decomposition stages, the STA449F5 Jupiter^®^ thermobalance from NETZSCH (Weimar, Germany) was used. The equipment offers high resolution, a wide temperature range, and low balance drift. The samples were placed in standard 85 µL alumina (Al_2_O_3_) crucibles with an average weight of between 5 and 7 mg. For the tests, mass flow controllers (MFCs) used oxygen and argon at rates of 252.5 mL/min and 249.3 mL/min, respectively. These were subjected to a heating program from 25 to 600 °C with a heating rate of 10 °C/min in a nitrogen atmosphere.

#### 2.3.2. Mechanical Characterization

The mechanical characterization was conducted through tensile, impact, and hardness tests. Tensile tests were performed using an ELIB 30 universal testing machine (S.A.E. Ibertest, Madrid, Spain) equipped with a 5 kN load cell. A minimum of five different specimens were tested at a speed of 5 mm/min at room temperature. For the tensile test, the parameters specified in the ISO 527-1:2019 standard were used [22]. To evaluate the energy absorbed by the material during impact, tests were performed using a 6 J Charpy pendulum (Metrotec, San Sebastian, Spain). Five measurements were taken with unnotched bending-type specimens, following the parameters defined in the ISO 179-1:2023 standard [23]. Finally, surface hardness was assessed by taking five measurements at different points on the surface of several specimens. A Shore D scale hardness tester (JBA S.A. model 673-D, Instruments JBot, S.A. Cabrils, Barcelona, Spain) was used, following the parameters outlined in the ISO 868:2003 standard [24].

#### 2.3.3. FTIR Analysis

Attenuated Total Reflectance–Fourier Transform Infrared Spectroscopy (FTIR-ATR) was performed using a Perkin Elmer Spectrum Two FT-IR spectrometer (PerkinElmer, Waltham, MA, USA), equipped with a universal ATR accessory. The analysis aimed to identify the functional groups of BPO and MA and in the various PLA/PCL blend samples. FTIR spectra were recorded within a wavenumber range of 4000 to 600 cm^−1^, with a resolution of 4.0 cm^−1^ and an interval of 1.0 cm^−1^.

#### 2.3.4. Determination of Surface Wettability by Contact Angle Measurements

The wettability of the surface was determined through the water contact angle (WCA) on the surface of the PLA samples using the sessile drop method. At least six drops of water (≈ 1.5 µL) were randomly deposited on the surface of the sample with a precision syringe at room temperature, and the water contact angle was measured eight times for each drop at room temperature using an EasyDrop-FM140 optical goniometer from Kruss Equipments (Hamburg, Germany).

#### 2.3.5. Morphological Characterization of the Specimens

The specimens were morphologically characterized after the Charpy impact test using Zeiss Ultra 55 model field emission scanning electron microscopes (FESEMs) (Oxford Instruments, Pleasanton, CA, USA) operating at an accelerating voltage of 2 kV. To improve surface conductivity, the samples were coated with a gold layer using an Emitech SC7620 Sputter Mod Coater, from Quorum Technologies (East Sussex, UK).

## 3. Results

### 3.1. FTIR Results

**Figure 2:**
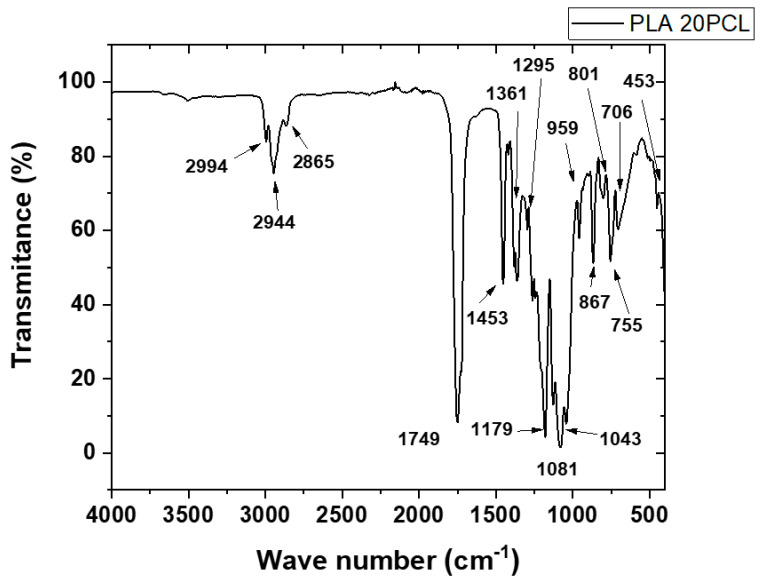
FTIR curve of PLA/PCL blend.

This FTIR spectrum corresponds to a polymeric blend composed of polylactic acid (PLA) and polycaprolactone (PCL) (see Figure 2), showing the characteristic absorption bands associated with the functional groups of both polymers. In the region around 2994 cm^−1^ and 2944 cm^−1^, we observe stretching vibrations of the –CH_3_ and –CH_2_ groups, typical of the aliphatic chains present in both PLA and PCL. The peak at 2865 cm^−1^ is also related to symmetric –CH_2_ stretching. A strong and sharp peak at 1749 cm^−1^ is attributed to the carbonyl (C=O) stretching vibration, which is prominent in both ester-based polymers. The band at 1453 cm^−1^ is associated with bending vibrations of the –CH_2_ and –CH_3_ groups. The absorption near 1361 cm^−1^ and 1295 cm^−1^ corresponds to C–H bending, particularly from PLA. The peaks at 1179 cm^−1^ and 1081 cm^−1^ are due to C–O–C stretching vibrations, also indicative of ester linkages. The region between 959 cm^−1^ and 756 cm^−1^ shows complex deformation and bending modes associated with the skeletal vibrations of the polymer backbone. Notably, the bands at 867 cm^−1^ and 786 cm^−1^ are often related to PCL-specific modes. Finally, the band at 1043 cm^−1^ corresponds to C–O stretching, further confirming the presence of ester functionalities in the blend. This spectral analysis confirms the coexistence of PLA and PCL within the blend through the identification of their characteristic functional groups.

**Figure 3:**
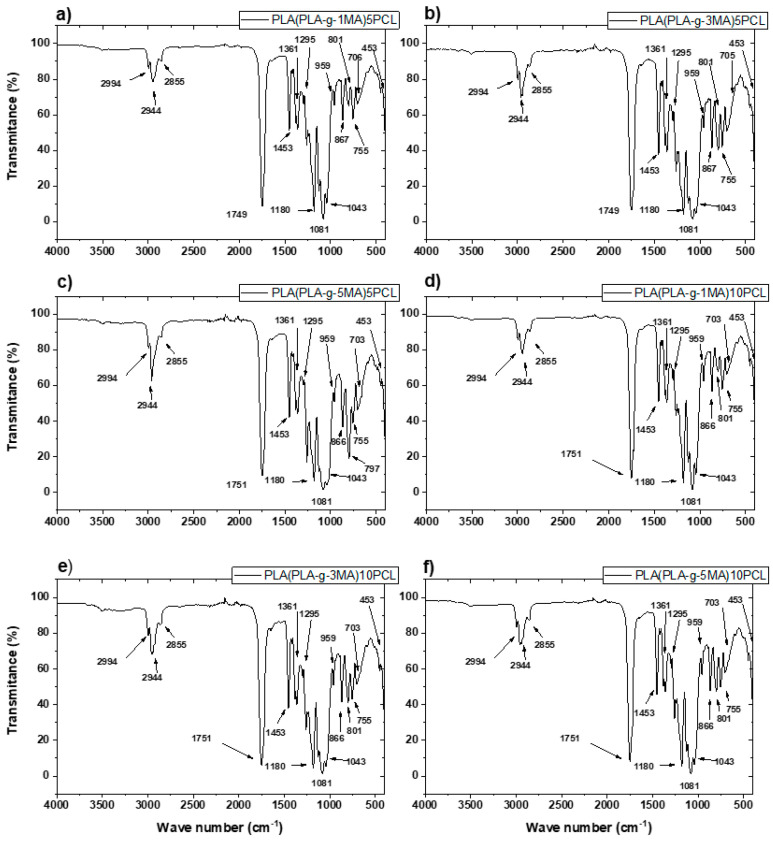
FTIR curves of PLA/PCL blends: (**a**) PLA (PLA-g-1MA)5 PCL; (**b**) PLA (PLA-g-3MA)5 PCL; (**c**) PLA (PLA-g-MA)5 PCL; (**d**) PLA (PLA-g-1MA)10 PCL; (**e**) PLA (PLA-g-3MA)10 PCL; and (**f**) PLA (PLA-g-MA)10 PCL.

The FTIR spectra obtained from the PLA-g-MA/PCL formulations revealed several characteristic absorption bands corresponding to the chemical structures of poly (lactic acid) (PLA), polycaprolactone (PCL), and maleic anhydride (MA) (see Figure 3). Furthermore, a detailed examination was carried out to assess the possible presence of residual benzoyl peroxide (BPO), which was used as the radical initiator during reactive extrusion.

Firstly, the main peaks associated with the PLA structure were clearly observed across all spectra. The most prominent band appeared near 1750 cm^−1^, corresponding to the C=O stretching of the ester group in PLA. Additionally, peaks were identified around 2995 cm^−1^ and 2945 cm^−1^, and were attributed to the C–H stretching vibrations of CH_3_ and CH units, respectively. Bending vibrations were seen near 1455 cm^−1^, 1385 cm^−1^, and 1355 cm^−1^, consistent with CH_3_ and CH deformation modes [25,26].

In the region from 1185 to 1045 cm^−1^, several peaks were assigned to the asymmetric and symmetric stretching of C–O–C ester groups. A band at 865 cm^−1^ was also present, corresponding to C–COO deformation, commonly reported for PLA.

In addition to the PLA signals, characteristic PCL bands were also evident. The C=O stretching of PCL ester groups appeared near 1720–1730 cm^−1^, partially overlapping with the carbonyl signal of PLA. The C–H stretching from methylene groups in PCL was observed around 2945 cm^−1^ and 2865 cm^−1^. Vibrational modes near 1295 cm^−1^ and 1240 cm^−1^ were attributed to C–O and C–C stretching, while signals between 1160 and 1100 cm^−1^ reflected C–O–C bending, all of which are consistent with PCL’s aliphatic ester structure.

Regarding the grafting of maleic anhydride onto PLA, the presence of MA was inferred from subtle but distinct spectral features. In particular, the carbonyl region around 1750 cm^−1^ showed broadening and increased intensity in the PLA-g-MA samples when compared to neat PLA. This effect is due to the superimposition of the carbonyl bands from both PLA and the anhydride groups of MA. In some spectra, especially for PLA-g-3MA and PLA-g-5MA formulations, additional absorption bands were observed between 1885 and 1850 cm^−1^. These bands are attributed to asymmetric C=O stretching of anhydride functionalities, supporting the successful grafting of MA onto PLA chains. These findings are consistent with prior reports in the literature, such as the work of Orozco et al., who noted that the spectral features of MA are often subtle due to its low concentration, yet detectable through carbonyl shifts and minor absorptions in this range.

A critical part of the analysis focused on determining the presence of residual benzoyl peroxide (BPO), which would indicate incomplete decomposition during processing. BPO has several characteristic FTIR signals, most notably the O–O peroxide bond stretching near 880–890 cm^−1^. Aromatic ring vibrations appear at 1600 cm^−1^, 1500 cm^−1^, and 1450 cm^−1^, while aromatic C–H stretching is found in the 3100–3050 cm^−1^ region. Additionally, the C=O stretching of benzoate groups from BPO can overlap with PLA and PCL near 1720–1760 cm^−1^, making this region less definitive for BPO identification.

After carefully inspecting the FTIR spectra, no significant absorption was observed in the 880–890 cm^−1^ region, indicating the absence of the O–O bond and, therefore, no detectable residual BPO. Similarly, no peaks were observed around 1600, 1500, or 1450 cm^−1^ that could be attributed to the aromatic vibrations of BPO. The carbonyl band at 1750 cm^−1^ is consistent with the ester groups in PLA, PCL, and MA, and cannot by itself confirm the presence of BPO. However, in the absence of aromatic-related peaks, it is unlikely that BPO remains in formulation. These observations suggest that BPO was fully decomposed during melt processing at 180 °C, which is above the thermal decomposition range of BPO (typically between 80 and 100 °C).

In conclusion, the FTIR spectra confirmed the presence of key functional groups associated with PLA, PCL, and grafted MA, supporting the successful preparation of PLA-g-MA/PCL blends. No residual BPO was detected, as evidenced by the absence of characteristic peroxy and aromatic bands in all the spectra. These results indicate that the extrusion conditions applied (a temperature profile from 170 to 185 °C and a 100 rpm screw speed) were sufficient to promote the complete decomposition of BPO, ensuring clean grafting reactions without initiator residue. This aligns with findings in related studies where FTIR proved effective in monitoring MA grafting and confirming the elimination of peroxide residues after processing.

### 3.2. Thermal Properties

The glass transition temperature (T_g_) can be used to assess thermal properties. Additionally, variations in cold crystallization temperature (T_cc_), melting temperature (T_m_), and the percentage of crystallinity (X_C_) are often indicative of the interactions between different components [27]. The DSC results obtained are summarized in Table 3. To ensure accurate assessments, the thermal data was analyzed based on the second heating run, which eliminates any potential influence of process history.

The thermal behavior of the PLA/PCL blends modified with different concentrations and types of maleic anhydride (MA)-grafted PLA (PLA-g-MA) was studied using differential scanning calorimetry (DSC). The results reveal how varying the compatibilizer type and amount influences the thermal transitions and crystallinity of the blends.

The unmodified blend, composed of 80% PLA and 20% PCL (PLA 20PCL), exhibited a melting temperature (T_m_) of 154.1 °C and a cold crystallization temperature (T_cc_) of 127.1 °C. Despite showing moderate values for the enthalpy of cold crystallization (ΔH_cc_ = 14.9 J/g) and melting enthalpy (ΔH_m_ = 15.3 J/g), the degree of crystallinity (X_c_) was extremely low (0.4%), suggesting poor crystallization under the experimental conditions.

Incorporating 5 wt.% of PLA-g-1MA into the PLA/PCL matrix led to a slight decrease in both T_m_ (151.1 °C) and T_cc_ (122.2 °C). However, a noticeable increase was observed in ΔH_m_ (20.3 J/g), resulting in a crystallinity of 5.5%, which suggests that PLA-g-1MA enhances the compatibility between PLA and PCL, promoting more effective crystallization [8]. Similarly, the use of 5 wt.% PLA-g-3MA produced T_m_ and T_cc_ values of 151.5 °C and 123.3 °C, respectively. The crystallinity in this case reached 3.6%, indicating moderate improvement compared to the unmodified blend.

When PLA-g-5MA was added at 5 wt.%, the melting and crystallization temperatures were 151.9 °C and 123.5 °C, respectively. While the ΔH_cc_ (18.6 J/g) and ΔH_m_ (19.1 J/g) values were relatively high, the resulting X_c_ remained low (0.5%). This suggests that although energy input was sufficient for crystallization, the steric hindrance introduced by the higher MA content may have limited chain mobility and thus reduced crystalline structure formation [8].

Increasing the content of PLA-g-1MA to 10 wt.% caused a further shift in thermal behavior. The melting temperature slightly decreased to 151.7 °C, while the T_cc_ remained stable at 122.1 °C. The higher ΔH_m_ (22.5 J/g) and ΔH_cc_ (19.7 J/g) values led to a crystallinity of 3.0%, which, although lower than the 5 wt.% version, still reflects the compatibilization effect. The use of 10 wt.% PLA-g-3MA showed similar thermal behavior (T_m_ = 151.5 °C, T_cc_ = 122.4 °C), with a ΔH_m_ of 19.5 J/g and crystallinity at 2.5%, confirming a moderate enhancement in thermal transitions.

The sample containing 10 wt.% PLA-g-5MA exhibited the lowest T_m_ (150.4 °C) and T_cc_ (120.0 °C) of all the formulations. Despite showing the highest enthalpy values (ΔH_cc_ = 21.2 J/g and ΔH_m_ = 21.4 J/g), its crystallinity remained low (1.2%), reinforcing the idea that excessive MA functionalization may hinder efficient crystal formation due to reduced chain flexibility and potential crosslinking effects.

In conclusion, the addition of PLA-g-MA compatibilizers, particularly PLA-g-1MA, significantly modifies the thermal behavior of PLA/PCL blends. Moderate concentrations (5–10 wt.%) enhance compatibility and crystallinity, with PLA-g-1MA showing the most promising results. In contrast, excessive MA content, especially in PLA-g-5MA, appears to limit crystallization despite increased thermal energy absorption. These findings support the use of functionalized PLA to tailor the thermal properties of biodegradable polymer blends.

**Figure 4:**
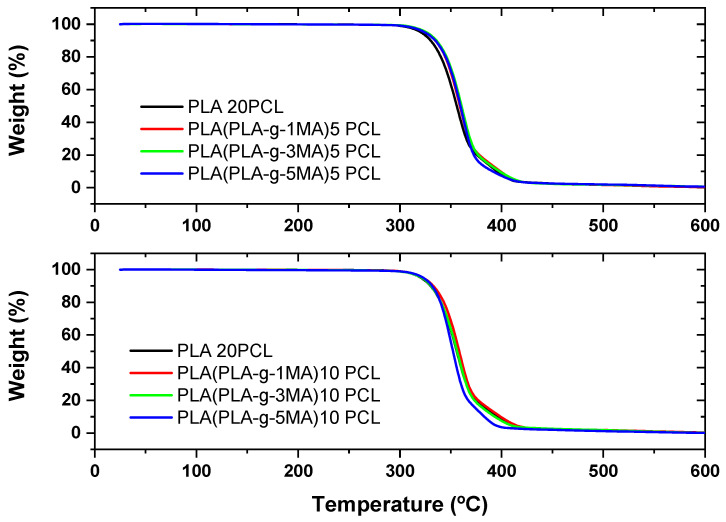
Thermal analysis (TGA) of PLA/PCL blends with different PLA-g-MA modifications.

**Figure 5:**
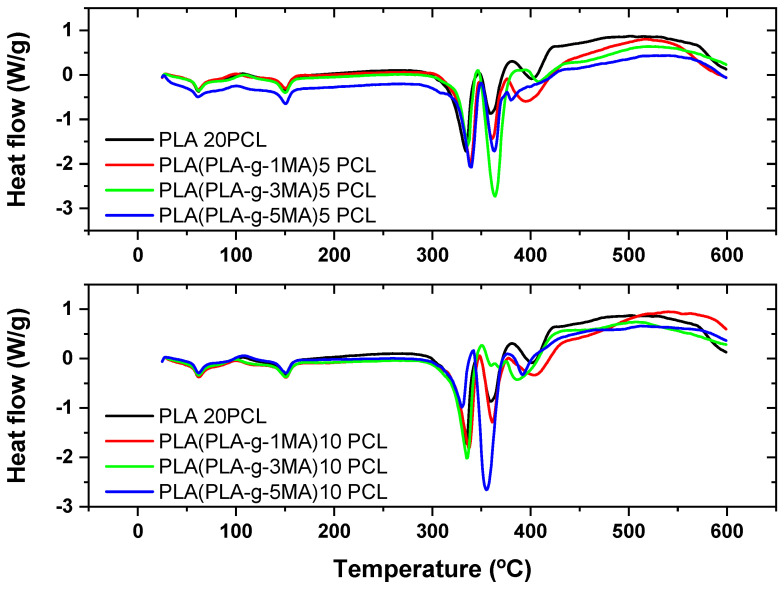
Thermal analysis (DSC) of PLA/PCL blends with different PLA-g-MA modifications.

Thermogravimetric analysis (TGA) and its derivative (DTG) reveal the thermal behavior of PLA/PCL formulations with different concentrations of MA as a compatibilizer (see Figure 4 and Figure 5). In PLA 20PCL, the main degradation occurs between 350 and 400 °C, with a single well-defined stage, indicating typical thermal degradation of PLA. When incorporating PLA-g-MA, variations in thermal stability are observed, with a slight decrease in the initial degradation temperature, possibly due to the presence of modified bonds that influence the polymer’s decomposition. In the PLA/PLA-g-1MA/5PCL formulation, thermal degradation follows a similar pattern to PLA 20PCL, although with slight variations in the DTG curve; this suggests that compatibilization does not drastically alter thermal stability. However, when increasing the amount of MA to 3MA and 5MA, the DTG curves show a greater dispersion of degradation peaks, indicating a more thermally heterogeneous structure, likely due to interactions between MA and PCL. The PLA/PLA-g-5MA/5PCL formulation exhibits slightly higher thermal stability compared to the other modifications, suggesting that a higher concentration of MA may improve the thermal resistance of the material. In conclusion, the addition of MA modifies the thermal degradation of the PLA/PCL system, with a tendency to generate more complex structures and potentially enhance thermal stability when higher MA concentrations are used.

In the PLA 20PCL formulation, the main degradation occurs in the 350–400 °C range, indicating that the addition of 10% PCL does not drastically alter the thermal stability of PLA. However, the presence of multiple peaks in the DTG curve suggests the occurrence of multi-step degradation processes. Upon incorporating PLA-g-1MA, the degradation temperature does not vary significantly, and the DTG curve retains a profile comparable to that of PLA 20PCL, suggesting that the compatibilizer does not substantially modify the overall thermal degradation behavior. In contrast, when increasing the amount of MA to 3MA and 5MA, a broader distribution of degradation peaks is observed, which indicates a more complex interaction between PLA, PCL, and MA, likely associated with morphological changes in the blend that influence its thermal response.

In particular, in the PLA/PLA-g-3MA/10PCL formulation, the DTG curve shows a broadening of the peaks, indicating that degradation occurs less uniformly, likely due to improved compatibility between the phases, which generates regions with different thermal resistance. On the other hand, the PLA/PLA-g-5MA/10PCL formulation exhibits a slight improvement in thermal stability, with a DTG curve displaying multiple well-defined peaks, suggesting a greater homogenization of the polymeric system and better interactions between PLA and PCL in the presence of 5MA.

Overall, it can be concluded that increasing the amount of MA alters the thermal degradation process, promoting greater compatibilization between PLA and PCL, although with a possible impact on the uniformity of thermal decomposition. The formulation with 5MA appears to be the most thermally stable among the options analyzed, making it an interesting candidate for applications where a balance between compatibilization and thermal resistance is required.

### 3.3. Mechanical Properties

**Figure 6:**
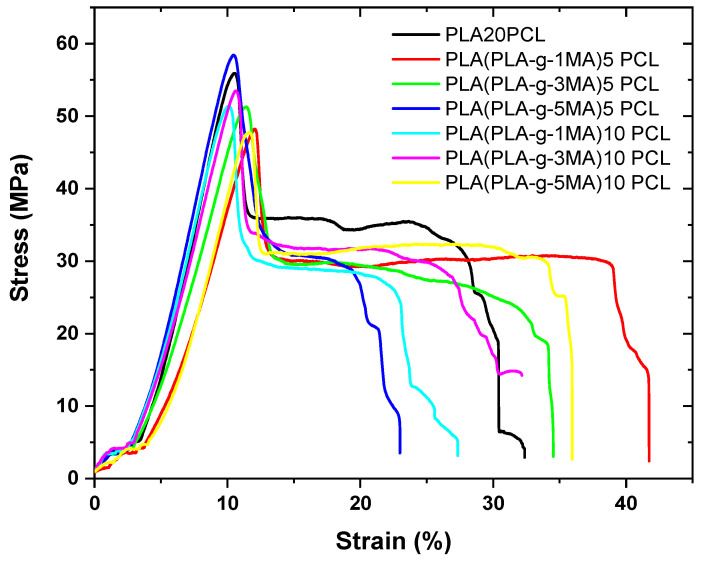
Strain–stress graph of different PLA/PCL Blends.

The analysis of mechanical properties is essential to understanding the structural behavior of polymeric materials and their suitability for specific applications. In this study, key parameters were evaluated, such as elongation at break, which reflects the material’s ability to deform before fracturing; maximum shear stress, which relates to the material’s resistance to mechanical loads; as well as impact strength and Shore D hardness, which provide insights into the material’s toughness and surface rigidity, respectively. These tests, together with the stress–strain graph shown below, allow for the assessment of how PLA modification with PCL and PLA-g-MA affects the mechanical performance of the system, offering a comprehensive view of its ductility, structural strength, and stability under impact deformation. The stress–strain graph is shown below (see Figure 6).

**Figure 7:**
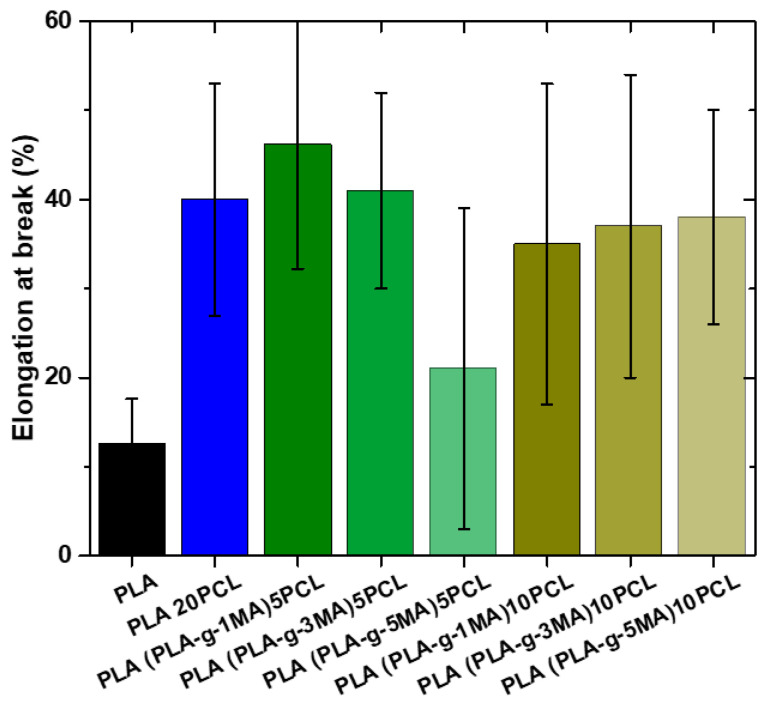
Elongation at break values of different PLA/PCL Blends.

Pure PLA exhibits a very low elongation at break (12.6% ± 5), indicating its brittle nature (see Figure 7). In contrast, PLA 20PCL shows a significant improvement (40% ± 13), suggesting that the addition of PCL increases the material’s ductility.

In formulations containing PLA-g-MA, the elongation at break varies depending on the percentage used. The 5 PLA-g-MA formulation shows values of 46.2% ± 13, 40.7% ± 11, and 20.9% ± 20, indicating a general improvement in ductility compared to pure PLA, but with a high standard deviation, suggesting substantial variability in the results. In the case of 10 PLA-g-MA, values range between 20% ± 11 and 38.4% ± 12, with an intermediate value of 37% ± 17.6. This suggests that a higher PLA-g-MA content does not necessarily lead to a consistent increase in elongation at break and that the material may exhibit heterogeneous behavior.

Overall, the addition of PCL significantly improves ductility, while PLA-g-MA also increases elongation at break, although with greater variability, which could indicate inconsistencies in the material’s structure or in phase dispersion.

**Figure 8:**
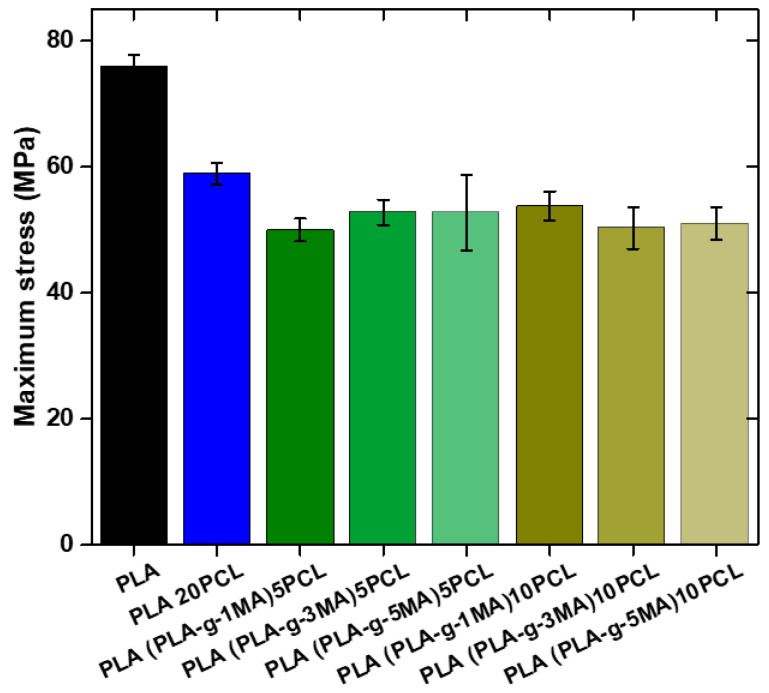
Maximum stress values of different PLA/PCL Blends.

Pure PLA continues to show the highest maximum strength (76 MPa ± 1.8), while PLA 20PCL exhibits a significant reduction (59 MPa ± 1.7), suggesting a decrease in stiffness due to the addition of PCL (see Figure 8).

In the formulations containing PLA-g-MA, variations in strength are observed depending on the percentage used. The 5 PLA-g-MA formulation shows values of 49.7 MPa ± 1.8, 52.8 MPa ± 2, and 52.7 MPa ± 5, indicating a decrease compared to pure PLA, although with relatively stable behavior across the different measurements. In the case of 10 PLA-g-MA, the values range from 53.8 MPa ± 2.8 to 50.3 MPa ± 3.5 and 50.77 MPa ± 2.5, suggesting that variability increases with a higher PLA-g-MA content.

Overall, the addition of PLA-g-MA reduces strength compared to pure PLA, although some values suggest a slight improvement over PLA 20PCL. Furthermore, the standard deviation increases with higher PLA-g-MA concentrations, indicating greater dispersion in the obtained values and possibly lower homogeneity in the material’s microstructure.

**Figure 9:**
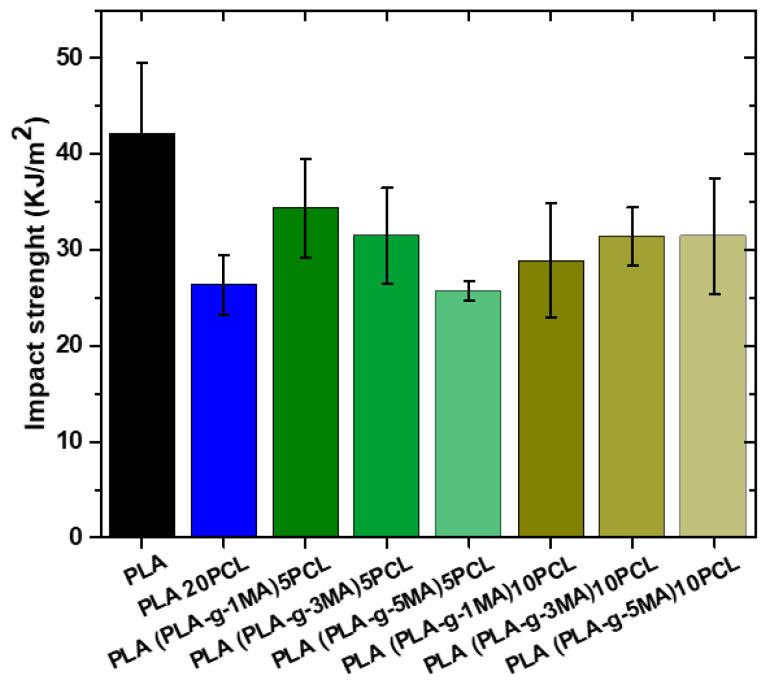
Impact strength values of different PLA/PCL Blends.

The impact test results show a clear trend in the resistance of PLA and its modifications (see Figure 9). Pure PLA exhibits the highest impact strength value at 42.1 KJ/m^2^, with a standard deviation of 7, indicating moderate variability in the results. However, when 20% PCL is added, the impact strength decreases significantly to 26.3 KJ/m^2^, with a lower standard deviation of 3, suggesting a more homogeneous blend but with a lower impact absorption capacity. When 5% PLA-g-MA is introduced, an increase in impact strength to 34 KJ/m^2^ is observed, with standard deviations of 5 and 6, indicating a partial recovery of the mechanical properties lost with the addition of PCL. This suggests that compatibilization with PLA-g-MA helps improve stress transfer between the material phases, although the original strength of pure PLA is still not reached. On the other hand, with 10% PLA-g-MA, impact values show greater variability, with records of 29 and 31 KJ/m^2^ and standard deviations of 6.5 and 3. This dispersion indicates that phase interaction remains a determining factor and that compatibilization does not fully achieve homogeneity in impact resistance.

In general, it can be concluded that the incorporation of PCL significantly reduces the impact resistance of PLA, likely due to a decrease in the material’s stiffness. However, the introduction of PLA-g-MA partially improves resistance, indicating that it acts as a compatibilizing agent by enhancing phase adhesion. Despite this improvement, the dispersion in values suggests that compatibilization is not entirely effective and that there may be regions with differences in phase structure and distribution.

The Shore D hardness results obtained reflect relatively low variability in most measurements (see Table 4), with values ranging from 72.0 to 75.5, indicating that the material maintains moderately consistent hardness. The average value is approximately 74.3, suggesting that the analyzed material exhibits relatively high hardness within this category of polymers. Looking at it in more detail, the lowest recorded value is 72.0, with a standard deviation of 2, implying lower hardness in that specific measurement, although still within an acceptable range. On the other hand, the highest value obtained is 75.5, with a deviation of 0.58, indicating high uniformity in that measurement and more stable hardness. The remaining samples show intermediate values, such as 73.5 with a deviation of 3, which suggests greater variability in that specific sample, and 74.7 with a deviation of 2.6, indicating a tendency to maintain hardness around that value, albeit with some dispersion in the results. 

Overall, the material displays moderately high and stable hardness, with some variations in certain samples that may be attributed to differences in the internal structure of the material, testing methodology, or the distribution of its components.

### 3.4. Determination of Surface Wettability by Contact Angle

**Figure 10:**
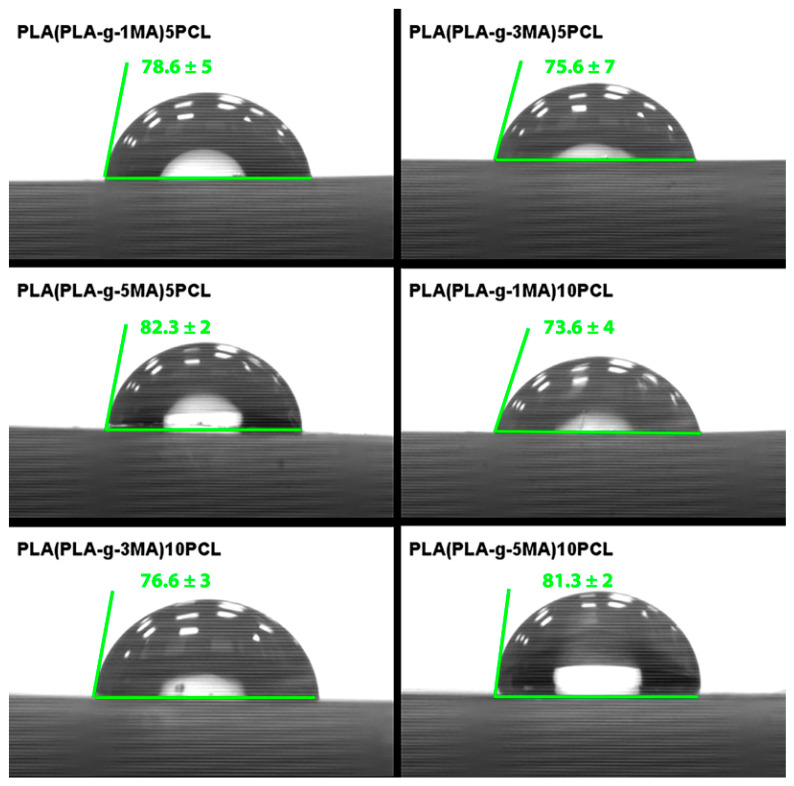
Contact angle results of PLA/PCL Blends.

The contact angle measurements were carried out on the PLA/PCL blends incorporating different proportions of PLA grafted with maleic anhydride (PLA-g-MA), aiming to assess the hydrophilicity of the surface in relation to the composition (see Figure 10). The results show clear influences of the grafting level and the PCL content on the wettability of the samples. The formulation PLA (PLA_g_1MA) 5PCL presented an average contact angle of 78.6°, with a standard deviation of 5, indicating a relatively moderate hydrophilicity. In comparison, PLA (PLA_g_3MA) 5PCL showed a slightly lower average contact angle of 75.6° and a higher standard deviation of 7.02, suggesting more surface irregularity or phase separation. On the other hand, PLA (PLA_g_5MA) 5PCL achieved the highest average contact angle among the 5 wt.% PCL blends, at 82.3°, with a low standard deviation of 2, pointing to better surface uniformity and reduced wettability. When the PCL content was increased to 10 wt.%, similar trends were observed. PLA (PLA_g_1MA) 10PCL had an average contact angle of 73.67° (SD: 4), PLA (PLA_g_3MA) 10PCL yielded 76.6° (SD: 3), while PLA (PLA_g_5MA) 10PCL recorded 81.3° (SD: 2), reaffirming the correlation between higher grafting levels and reduced surface hydrophilicity. These findings suggest that higher levels of grafted MA on PLA increase surface hydrophobicity, likely due to reduced polarity and improved interfacial adhesion with PCL. Overall, the contact angle data reflects the changes in surface energy induced by both the degree of functionalization and the PCL content, providing insight into the tuning of wettability for tailored applications in packaging or biomedical fields.

### 3.5. Morphological Characterization

**Figure 11:**
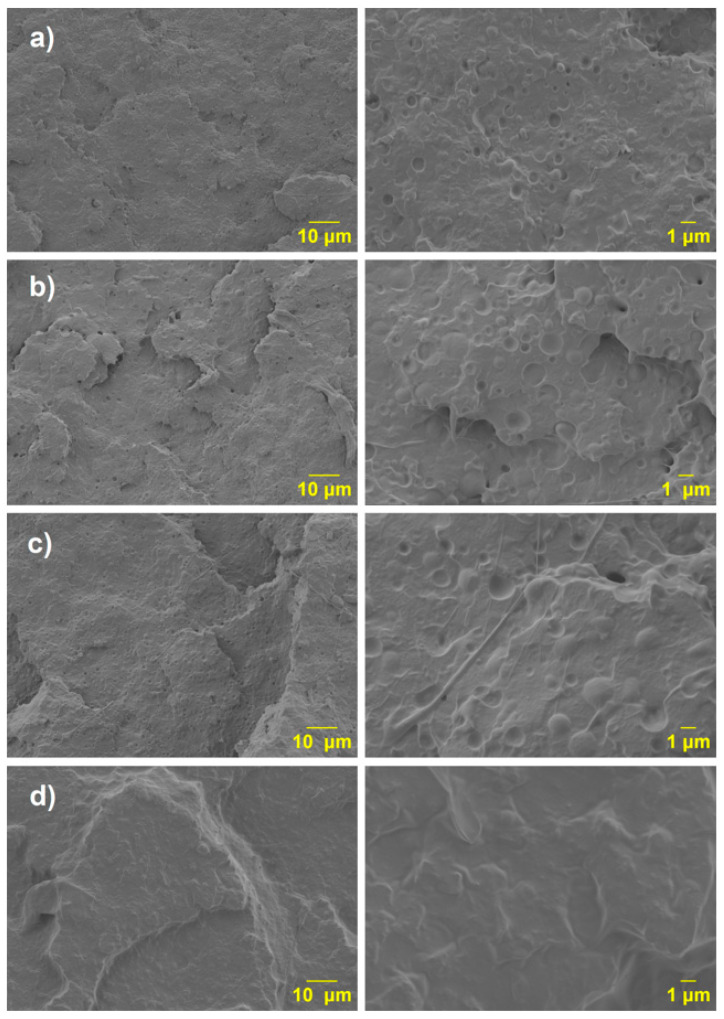
SEM micrographs of the blends: (**a**) PLA 20PCL; (**b**) PLA (PLA-g-1MA)5 PCL; (**c**) PLA (PLA-g-3MA)5 PCL; and (**d**) PLA (PLA-g-MA)5 PCL.

The SEM micrographs present the fractured surfaces of various PLA/PCL blends at two different magnifications (10 µm and 1 µm) (see Figure 11). These images provide insight into the morphological characteristics and the degree of compatibility between the PLA matrix and PCL phase, especially when modified with maleic anhydride (MA).

The first sample (Figure 11a), corresponding to the PLA/20PCL blend, shows a typical sea-island morphology, where discrete spherical PCL domains, with dimensions ranging between 1 and 3 μm, are dispersed in the PLA matrix. This morphology is characteristic of immiscible polymer blends, where one phase (in this case, PCL) is finely dispersed in the continuous phase (PLA). As reported in the literature [28], the viscosity of PCL at 180 °C is significantly lower than that of PLA, which results in a high viscosity ratio. This high viscosity contrast contributes to the formation of spherical domains during melt blending, as the lower-viscosity PCL tends to form droplets rather than co-continuous structures. Also, a clear phase separation is observed with PCL appearing as spherical domains without adhesion to the PLA matrix; voids are evident at the interface.

In the second sample (Figure 11b), PLA-(PLA-g-1MA)/5PCL, the size of the dispersed phase increases, but there is still no visible improvement in interfacial adhesion.

In the third sample (Figure 11c), corresponding to PLA-(PLA-g-3MA)/5PCL, the morphology appears smoother and more continuous. The voids are more uniform and less pronounced, which reflects an improved level of compatibility. The higher amount of MA grafting (3MA) contributes to better phase adhesion and reduced interfacial tension between PLA and PCL. In this case, a reduction in the size of the PCL domains is not observed [29]. However, when increasing the PLA-g-MA content to 3 wt.%, the appearance of certain filamentous structures is evident. These features are likely due to the increased ductility of the system, suggesting that although domain refinement is not clearly achieved, the morphological changes reflect the enhanced deformability of the dispersed phase.

In the fourth sample (Figure 11d), PLA-(PLA-g-5MA)/5PCL, no distinct phase separation can be observed in the SEM micrographs. The morphology appears more homogeneous, with a continuous structure suggesting a high degree of compatibility between the PLA matrix and the PCL phase. The absence of visible domain boundaries indicates that the maleic anhydride grafting at 5 wt.% may have effectively enhanced the interfacial adhesion and molecular interactions between the two polymers. However, despite the lack of clear phase separation, the presence of discrete particles within the matrix is still noticeable. These particles are likely associated with the precipitation or phase segregation of excess maleic anhydride or unreacted grafting residues. This observation suggests that while compatibilization improves, there may be a limit beyond which additional grafting does not further refine the morphology and could instead lead to the formation of secondary structures or by-products.

**Figure 12:**
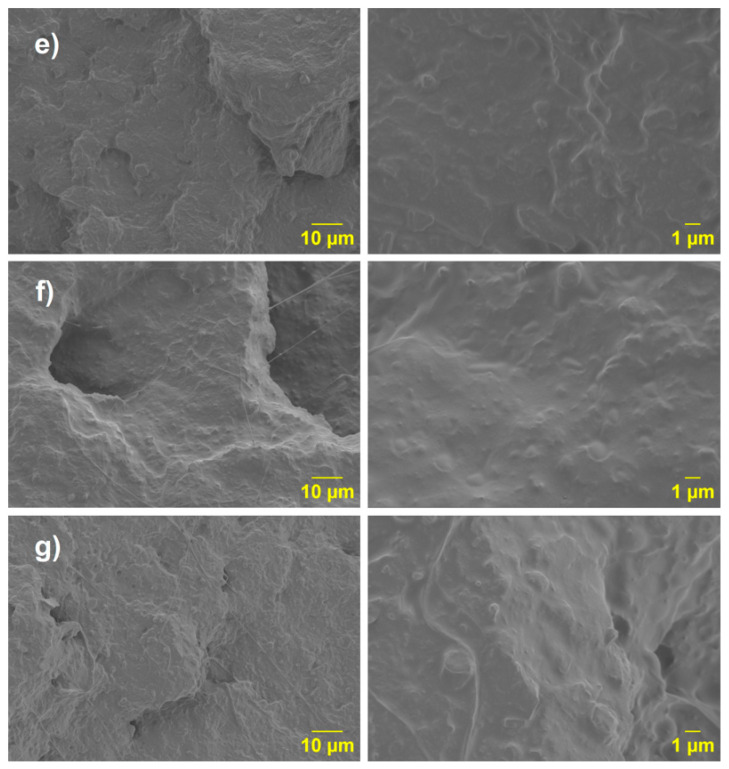
SEM micrographs of the blends: (**e**) PLA (PLA-g-1MA) 10PCL; (**f**) PLA (PLA-g-3MA)10 PCL; and (**g**) PLA (PLA-g-MA)10 PCL.

Finally, upon analyzing the PLA-(PLA-g-1MA)/10PCL, PLA-(PLA-g-3MA)/10PCL, and PLA-(PLA-g-5MA)/10PC SEM images (Figure 12), a morphology like the one described previously (Figure 11d) is observed.

## 4. Discussion

The PLA/PCL blend system remains a subject of intense investigation due to the inherently immiscible nature of the two polymers, which leads to weak interfacial adhesion and poor mechanical performance in unmodified blends. This study employed maleic anhydride-grafted PLA (PLA-g-MA) as a reactive compatibilizer and systematically evaluated its effect on the blend’s thermal, mechanical, morphological, and surface properties. The observed trends are cross compared with the published literature to critically contextualize the performance and validate the approach.

The thermal degradation behavior observed in TGA/DTG indicated that PLA/PCL (20/80) blends undergo degradation predominantly in a single stage between 350 and 400 °C, as is characteristic of PLA. With the addition of PLA-g-MA, particularly at 3MA and 5MA, the DTG curves showed peak broadening and multi-stage degradation. This suggests a more complex decomposition pathway, likely driven by improved interfacial mixing but increased phase heterogeneity. Similar trends were reported by [13], who noted that PLA-g-MA enhances interfacial interactions while increasing structural complexity. Ref. [30], using gamma irradiation, observed comparable morphological disruptions and increased surface irregularities linked to higher crosslinking, analogous in effect to what excessive grafting with MA might produce.

DSC analysis provided further insights. PLA-g-1MA at 5 wt.% increased crystallinity to 5.5%, confirming its efficacy in promoting better molecular ordering, which is consistent with the enhanced thermal resistance seen in TGA. However, higher MA contents (e.g., PLA-g-5MA) resulted in reduced crystallinity (< 1.5%), despite higher melting enthalpies, implying hindered chain mobility due to steric effects or localized crosslinking—observations mirrored in [31], where excessive compatibilizer (GMA) also led to reduced uniformity and molecular ordering. Thus, both the present and past findings highlight that while compatibilizers like MA or GMA initially enhance ordering and stability, they exhibit non-linear behavior, with over-compatibilization leading to performance deterioration due to disrupted crystallization dynamics.

From a mechanical standpoint, the elongation at break of the PLA/PCL blends increased significantly with the PCL addition (from 12.6% to 40%) and increased further with the addition of PLA-g-MA (up to ~46%). This enhancement in ductility indicates successful interfacial compatibilization, corroborated by [27], who demonstrated similar behavior with PEG/PPG-based block copolymers.

However, tensile strength consistently decreased upon PCL incorporation and showed limited recovery with PLA-g-MA. While some improvement over PLA 20PCL was observed, the values remained lower than those of neat PLA. This reduction is likely due to reduced crystallinity (as confirmed by DSC), phase softening from PCL, and morphological inconsistencies. These results are consistent with those from [16], who found that although impact strength significantly improved with GMA/acrylate-based compatibilizers, the tensile modulus and yield strength diminished at higher compatibilizer contents.

Ref. [13] reported similar trends with PLA-g-MA, where enhanced ductility was achieved without compromising stiffness, but only at moderate grafting levels (~0.7 wt.% MA). Beyond that, structural integrity suffered, reaffirming that compatibilizer concentration plays a critical role in balancing toughness and strength.

FTIR spectra confirmed the successful grafting of MA onto the PLA backbone, as evidenced by carbonyl band broadening and new absorptions around 1850–1885 cm^−1^. These features correspond to anhydride groups and align with the spectral markers described by [13,31]. Importantly, the absence of characteristic benzoyl peroxide (BPO) peaks suggests that the reactive extrusion conditions (170–185 °C) were effective in initiating grafting without leaving initiator residue, ensuring clean chemical functionalization.

The incorporation of PLA-g-MA improves the compatibility between PLA and PCL, as shown in the SEM micrographs (Figure 9 and Figure 10). In the uncompatibilized PLA/20PCL blend, a typical sea-island morphology with poor interfacial adhesion is observed. With 1 wt.% MA, no significant improvement is seen; however, at 3 wt.%, the morphology becomes more uniform and continuous, indicating better compatibility and increased ductility, even though domain size remains unchanged. At 5 wt.% MA, a homogeneous structure without visible phase separation is achieved, though some discrete particles may result from excess MA or unreacted residues. Blends with higher PCL content (10 wt.%) show similar morphologies, confirming that MA grafting enhances interfacial adhesion, though excessive amounts may lead to secondary structures.

The contact angle measurements reflected a direct correlation between MA content and surface hydrophobicity. Higher PLA-g-MA concentrations (5MA) led to increased contact angles (~82°) and lower deviations, indicating enhanced surface uniformity and reduced polar group exposure. These results parallel those from [26], who reported that increasing compatibilizer content reduced surface polarity and improved blend homogeneity.

Surface analysis also provides indirect evidence of interfacial adhesion quality. Ref. [32] emphasizes that improved surface uniformity in PLA/PCL systems is a marker of better morphological integration, which supports mechanical strength and thermal reliability.

Overall, the present study confirms that PLA-g-MA is a viable compatibilizer for PLA/PCL blends, capable of improving ductility, interfacial bonding, and thermal behavior when used in optimal proportions (e.g., 5 wt.%). Excessive concentrations (e.g., 10 wt.%) may deteriorate performance by limiting chain mobility, reducing crystallinity, and creating morphological inconsistencies. This trend is mirrored across other studies employing different compatibilizers (e.g., GMA, block copolymers), reinforcing the nonlinear and formulation-sensitive nature of reactive blending strategies. Ref. [16] further demonstrated that compatibilizer effectiveness is highly dependent on blend ratios—70/30 PLA/PCL showed the best performance—suggesting that not only type but also composition and processing parameters (temperature, shear rate) must be optimized. This aligns with [29], who showed that even non-chemical approaches like gamma irradiation require precise dose control to avoid degradation or over-crosslinking.

This study provides an integrated evaluation that combines thermal, mechanical, morphological, and surface analyses, offering a more comprehensive understanding of the compatibilization process. Unlike previous works that mainly focused on isolated properties, our approach highlights the interrelation between different performance aspects, allowing a clearer identification of the benefits and limitations of MA grafting. This holistic perspective constitutes a distinctive contribution to the optimization of PLA/PCL systems.

## 5. Conclusions

This study demonstrated that maleic anhydride-grafted PLA (PLA-g-MA) effectively enhances the thermal and mechanical performance of PLA/PCL biocomposites. The incorporation of 5–10 wt.% PLA-g-MA led to improved phase compatibility, particularly at moderate MA contents (1–3%), as evidenced by the increased elongation at break and impact strength. Thermal analysis (DSC and TGA) confirmed that PLA-g-1MA promotes greater crystallinity and slightly improves thermal stability, while higher MA concentrations (e.g., PLA-g-5MA) tend to reduce crystallinity and increase variability in thermal degradation, likely due to restricted chain mobility or morphological heterogeneity.

Mechanical testing revealed that although the addition of PCL significantly increases ductility, it decreases tensile strength. The inclusion of PLA-g-MA partially recovers impact resistance and elongation, but at the expense of increased dispersion in the results, especially at higher compatibilizer concentrations. The SEM analysis supported these findings, showing morphological changes consistent with enhanced interfacial adhesion and phase dispersion.

Additionally, the contact angle measures indicated that higher MA-grafting levels increase surface hydrophobicity and uniformity. FTIR confirmed the successful incorporation of MA into the PLA matrix and the complete decomposition of benzoyl peroxide (BPO) during processing, ensuring clean grafting reactions.

Overall, PLA-g-MA emerges as a viable compatibilizer for PLA/PCL systems. However, its concentration must be carefully controlled, as excessive levels can hinder crystallization and uniform mechanical behavior. The optimal balance between mechanical enhancement and thermal stability was found at 5 wt.% PLA-g-MA with 1–3 wt.% MA, suggesting a promising strategy for improving the performance of biodegradable PLA/PCL composites.

## Figures and Tables

**Table 1:** Summary of PLA-grafted compositions according to the weight content (wt.%) of PLA and different proportions.

Material-Code	PLA (wt.%)	MA (phr)	BPO (phr)
PLA 1g-MA	100	1	0.1
PLA 3g-MA	100	3	0.1
PLA 5g-MA	100	5	0.1

**Table 2:** Summary of compositions according to the weight content (wt.%) of PLA and PCL.

Material-Code	PLA (wt.%)	PCL (wt.%)	PLA g-1MA (wt.%)	PLA g-3MA (wt.%)	PLA g-5MA (wt.%)
PLA 20PCL	80	20	-	-	-
PLA (PLA-g-1MA) 5 PCL	75	20	5	-	-
PLA (PLA-g-3MA) 5 PCL	75	20	-	5	-
PLA (PLA-g-5MA) 5 PCL	75	20	-	-	5
PLA (PLA-g-1MA) 10 PCL	70	20	10	-	-
PLA (PLA-g-3MA) 10 PCL	70	20	-	10	-
PLA (PLA-g-5MA) 10 PCL	70	20	-	-	10

**Table 3:** Thermal properties for neat PLA/PCL blend and MA formulations.

Material Code	T_m-PCL_ (°C)	T_cc-PLA_ (°C)	T_m-PLA_ (°C)	∆H_cc_ (J/g)	∆H_m_ (J/g)	X_c_ (%)
PLA 20PCL	57	127	154	14.9	15.3	0.4
PLA (PLA-g-1MA) 5 PCL	55	122	151	15.1	20.3	5.5
PLA (PLA-g-3MA) 5 PCL	56	123	151	16.3	19.7	3.6
PLA (PLA-g-5MA) 5 PCL	55	123	151	18.6	19.1	0.5
PLA (PLA-g-1MA) 10 PCL	56	122	151	19.7	22.5	3.0
PLA (PLA-g-3MA) 10 PCL	55	122	151	17.1	19.5	2.5
PLA (PLA-g-5MA) 10 PCL	54	120	150	21.2	21.4	1.2

**Table 4:** Shore D hardness of PLA/PCL blends modified with maleic anhydride.

Material-Code	Shore D Hardness
PLA 20PCL	70.0 ± 2
PLA (PLA-g-1MA)5 PCL	73.5 ± 3
PLA (PLA-g-3MA)5 PCL	75.2 ± 2
PLA (PLA-g-5MA)5 PCL	75.5 ± 1
PLA (PLA-g-1MA)10 PCL	72 ± 2
PLA (PLA-g-3MA)10 PCL	74.8 ± 2
PLA (PLA-g-5MA)10 PCL	74.7 ± 2

## Data Availability

The data presented in this study are available on request from the corresponding author.
